# Supplementary material for: Comparison and Validation of Rapid Molecular Testing Methods for Theranostic Epidermal Growth Factor Receptor Alterations in Lung Cancer: Idylla versus Digital Droplet PCR
Source: Int J Mol Sci. 2023 Oct 27;24(21):15684. doi: 10.3390/ijms242115684 (PMC10648419; doi:10.3390/ijms242115684)
Supplement: Supplementary file 1 [file ijms-24-15684-s001.zip › ijms-2676079 - Supplementary Figures S1-S5.pdf]

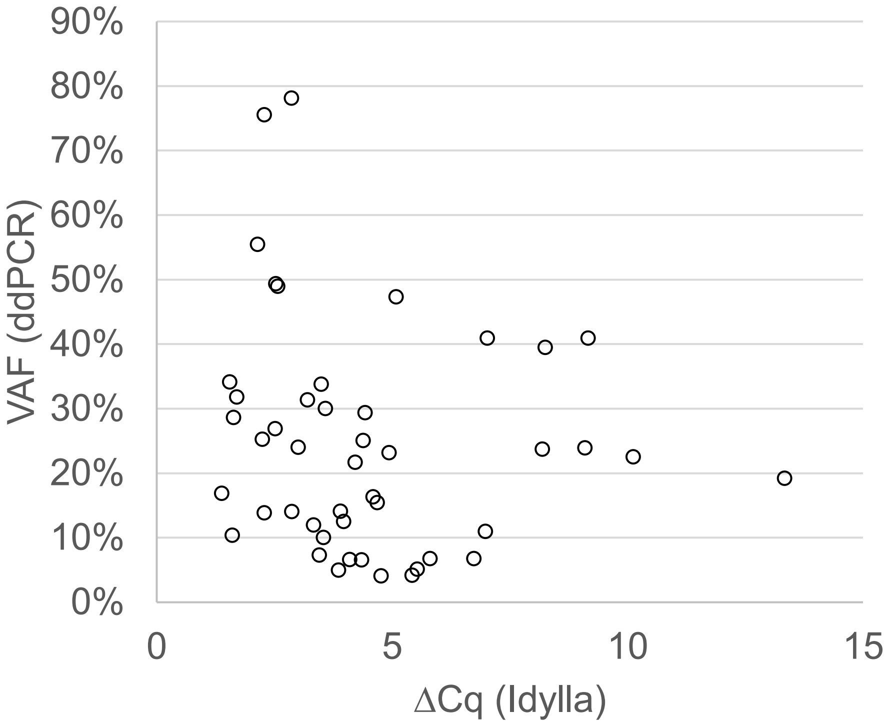

Figure S1. The estimated variant frequencies are not correlated

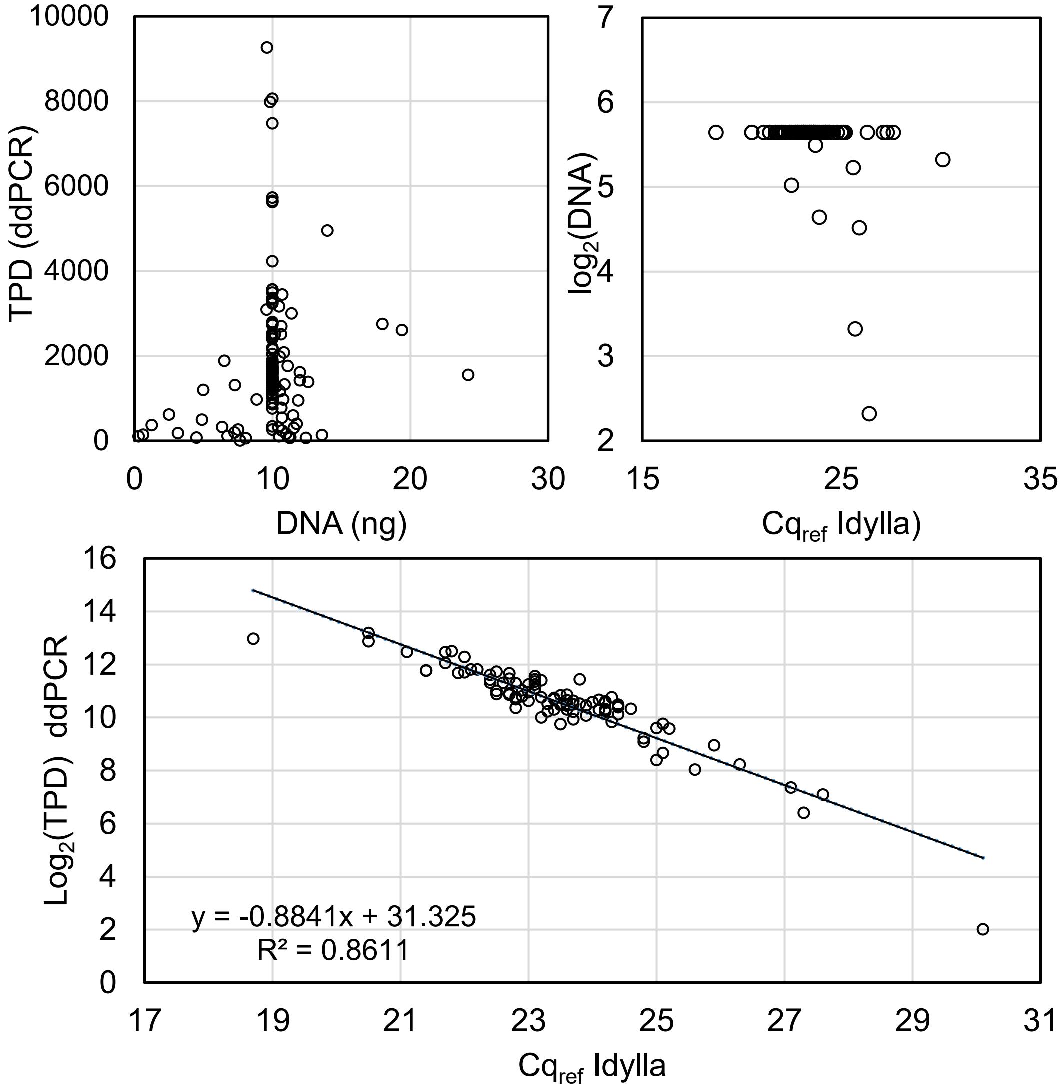

Figure S2. Robustness evaluation

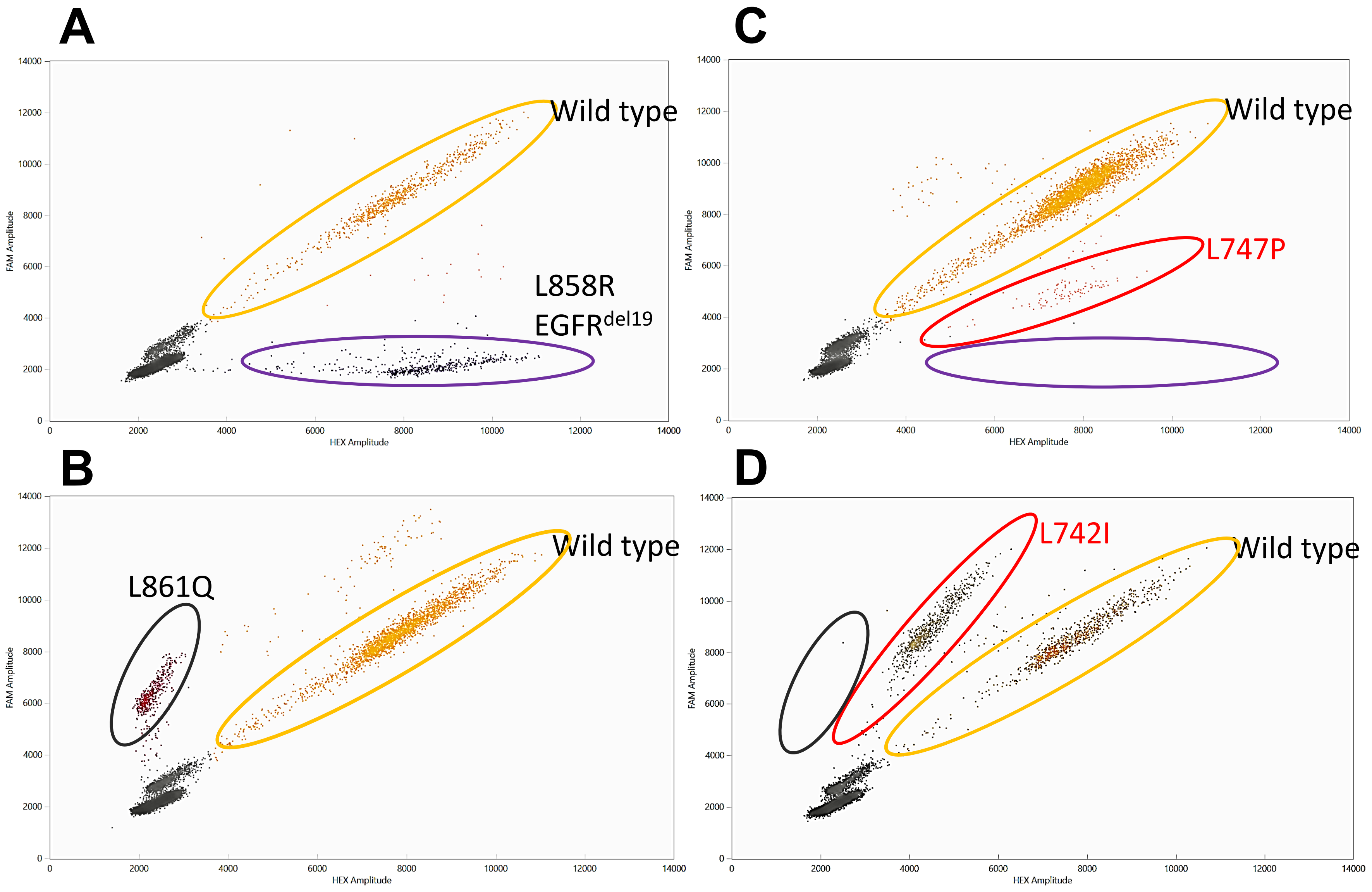

Figure S3. Results of ddPCR testing of SNP in position p.747 and p.742

Mutations detectable by the Idylla™ EGFR Mutation Assay

Exon	Mutation	DNA alteration
Exon 18	G719A	c.2156G > C
	G719C	c.2155G > T; c.2154_2155delisTT
	G719S	c. c.2155G > A
Exon 19	Del 9	c.2238_2248delinsGC; c.2239_2248delinsC; c.2240_2248del; c.2239_2247del
	Del 12	c.2239_2251delinsC; c.2240_2251del
	Del 15	c.2235_2249del; c.2236_2250del; c.2239_2253del; c.2240_2254del; c.2238_2252del; c.2237_2251del; c.2235_2252delinsAAT; c.2237_2252delinsT; c.2234_2248del; c.2236_2253delinsCTA; c.2237_2253delinsTA; c.2235_2251delinsAG; c.2236_2253delinsCAA;c.2230_2249delinsGTCAA
	Del 18	c.2240_2257del; c.2237_2255delinsT; c.2239_2256del; c.2236_2253del; c.2239_2258delinsCA; c.2237_2254del; c.2238_2255del; c.2237_2257delinsTCT; c.2236_2255delinsAT; c.2236_2256delinsATC; c.2237_2256delinsTT; c.2237_2256delinsTC;c.2235_2255delinsGGT
	Del 21	c.2238_2258del; c.2236_2256del
	Del 24	c.2253_2276del
Exon 21	L858R	c.2573T > G; c.2573_2574delinsGT; c.2573_2574delinsGA
	L861Q	c.2582T > A

Targeted mutations and alterations detected by IDEGFR(b) SENSI-v3-50 Kit for ddPCR assays

Exon	Mutation	DNA alteration	Cosmic ID
Exon 19	p.E746_A750del (1)	c.2235_2249del15	COSM6223
	p.E746_A750del (2)	c.2236_2250del15	COSM6225
	p.L747_P753>S	c.2240_2257del18	COSM12370
	p.E746_T751>I	c.2235_2252>AAT (complex)	COSM13551
	p.E746_T751del	c.2236_2253del18	COSM12728
	p.E746_T751>A	c.2237_2251del15	COSM12678
	p.E746_S752>A	c.2237_2254del18	COSM12367
	p.E746_S752>V	c.2237_2255>T(complex)	COSM12384
	p.E746_S752>D	c.2238_2255del18	COSM6220
	p.L747_A750>P	c.2238_2248>GC (complex)	COSM12422
	p.L747_T751>Q	c.2238_2252>GCA (complex)	COSM12419
	p.L747_E749del	c.2239_2247del9TTAAGAGAA	COSM6218
	p.L747_S752del	c.2239_2256del18	COSM6255
	p.L747_A750>P	c.2239_2248TTAAGAGAAG>C(complex)	COSM12382
	p.L747_P753>Q	c.2239_2258>CA (complex)	COSM12387
	p.L747_T751>S	c.2240_2251del12	COSM6210
	p.L747_T751del	c.2240_2254del15	COSM12369
	p.L747_T751>P	c.2239_2251>C(complex)	COSM12383
	p.L747_T751del	c.2238_2252del15	COSM23571
	p.L747_S752>Q	c.2239_2256>CAA	COSM12403
	p.E746_T751>V	c.2237_2252>T	COSM12386
	p.E746_T751>T	c.2236_2253> ACG	/
	p.L747_A750>P	c.2239_2250>CCC	/
	p.L747_K754>QL	c.2239_2261>CAATT	/
	p.E746_K754>EQHL	c.2238_2261>GCAACATCT	/
	p.E746_S752>EQ	c.2238_2256>GCAA	/
	p.E746_A750>QP	c.2236_2248>CAAC	COSM13557
	p.E746_T751>Q	c.2236_2253>CAA	COSM22999
Exon 21	p.L858R	c.2573-2574TG>GT	COSM6224
		c.2573T>G	COSM12979
	p. L861Q	c.2582T>A	COSM6213

Figure S4. List of del19 alterations predicted to be detected

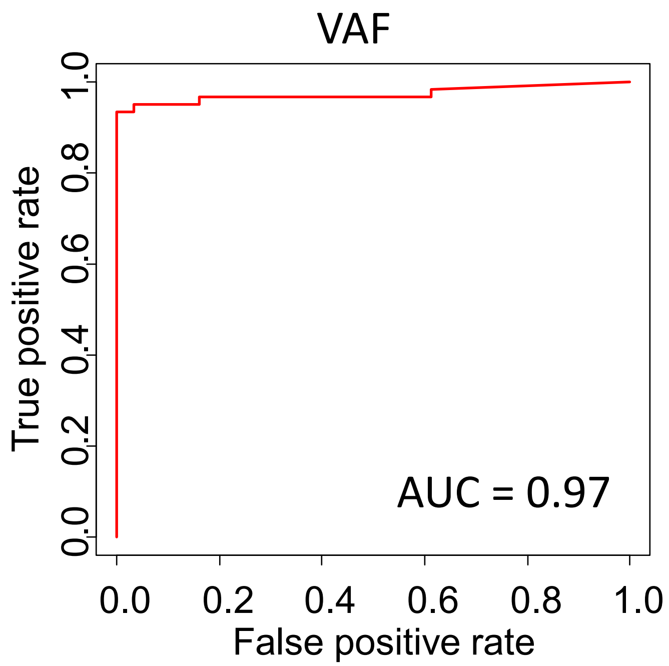

Figure S5. ROC curve analysed for VAF values obtained by ddPCR.
