# Supplementary material for: Comparison and Validation of Rapid Molecular Testing Methods for Theranostic Epidermal Growth Factor Receptor Alterations in Lung Cancer: Idylla versus Digital Droplet PCR
Source: Int J Mol Sci. 2023 Oct 27;24(21):15684. doi: 10.3390/ijms242115684 (PMC10648419; doi:10.3390/ijms242115684)
Supplement: Supplementary file 1 [file ijms-24-15684-s001.zip › Supplementary informations.pdf]

Supplementary informations

Table S1: Details of del19 sequences and frequencies among the TCGA and the local cohorts

Table S2: Detailed sample characteristics of the local database. This chart includes all information and results (genomic alterations, VAF, DNA concentration, Idylla, and ddPCR results) for the 122 samples included in this study.

Figure S1: the estimated variant frequencies are not correlated

Figure S2: Robustness evaluation

Figure S3: Results of ddPCR testing of SNP in position p.747 and p.742

Figure S4: List of del19 alterations predicted to be detected

Figure S5 : ROC curve analysed for VAF values obtained by ddPCR.
